# Design, Synthesis and Antifungal/Insecticidal Evaluation of Novel Cinnamide Derivatives

**DOI:** 10.3390/molecules16118945

**Published:** 2011-10-25

**Authors:** Yumei Xiao, Xiaoli Yang, Bo Li, Huizhu Yuan, Shuqing Wan, Yanjun Xu, Zhaohai Qin

**Affiliations:** 1 College of Science, China Agricultural University, Beijing 100193, China; 2 Lab of Insect Toxicology, South China Agricultural University, Guangzhou 510642, China; 3 Institute of Plant Protection, Chinese Academ y of Agriculture Science, Beijing 10193, China

**Keywords:** cinnamide, *β*-phenyl ethylamine, fungicidal activity, insecticidal activity

## Abstract

Twenty novel cinnamamide derivatives were designed and synthesized using as lead compound pyrimorph, whose morpholine moiety was replaced by *β*-phenylethylamine. All the compounds were characterized by their spectroscopic data. The fungicidal and insecticidal activities were also evaluated. The preliminary results showed that all the title compounds had certain fungicidal activities against seven plant pathogens at a concentration of 50 μg/mL, and compounds **11a** and **11l** showed inhibition ratios of up to 90% against *R. solani*. Most of the title compounds exhibited moderate nematicidal activities. In general, the morpholine ring may be replaced by other amines and a chlorine atom in the pyridine ring is helpful to fungicidal activity.

## 1. Introduction

**Figure 1:**
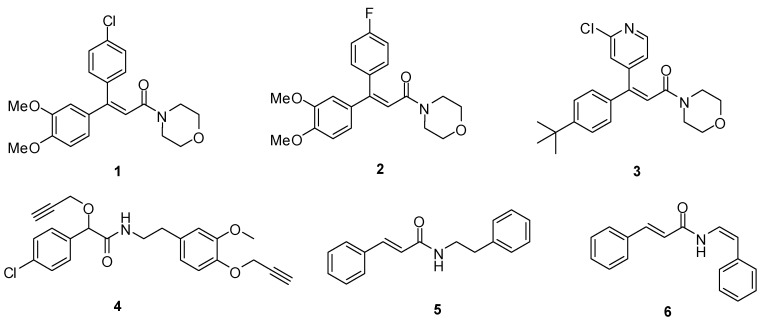
Several cinnamide and *β*-phenylethylamine derivitives with fungicide or insecticidal activity.

Cinnamides constitute an important class of compounds with a variety of biological properties, such as nervous central system depressant, anticonvulsant, muscle relaxant, antiallergic, antineoplastic and anti-infective activities, *etc*. [1,2,3,4,5,6,7]. In the agrochemical field, their avian repellent, fungicidal and herbicidal activities have also attracted the attention of many researchers [8,9], and several excellent cinnamide fungicides, for example dimethomorph (**1**) [10], fluormorph (**2**) [11] and pyrimorph (**3**) [12,13], have been successfully developed. Pyrimorph, containing a morpholine ring and a pyridine ring, is a novel fungicide developed by our lab that exhibited excellent activity against oomycetes [14,15]. *β*-phenylethylamines are also very important bioactive molecules that can be found in many natural and synthetic drugs. Mandipropamide (**4**), which controls foliar diseases caused by oomycetes, is a typical representative of *β*-phenylethylamines derivitives used in agrochemistry [16,17]. Wan *et al. * have reported the fungicidal activity of (*E*)-*N*-2-phenylethyl cinnamide (**5**) [18] and the insecticidal activity of lansiumamide B (**6**) against *B. xylophilus* and *Culex pipiens* have also been disclosed [19,20] (Figure 1).

Considering the important role of the cinnamoyl, pyridine ring and 2-phenylethylamine moieties in pesticides, their combination might result in novel bioactive molecules. In this study, using pyrimorph as lead compound, we have designed and synthesized a novel series of cinnamide derivatives in which the morpholine moiety in pyrimorph was replaced by a phenethylamino group. All of the target compounds were evaluated for fungicidal and nematicidal activity. Some title compounds showed good fugicidal activity at 50 μg mL^−1^, and the compound **11b** exhibited an LC_50_ of 113.8 μg mL^−1^ against *B. xylophilus*.

## 2. Results and Discussion

### 2.1. Synthesis

**Scheme 1:**
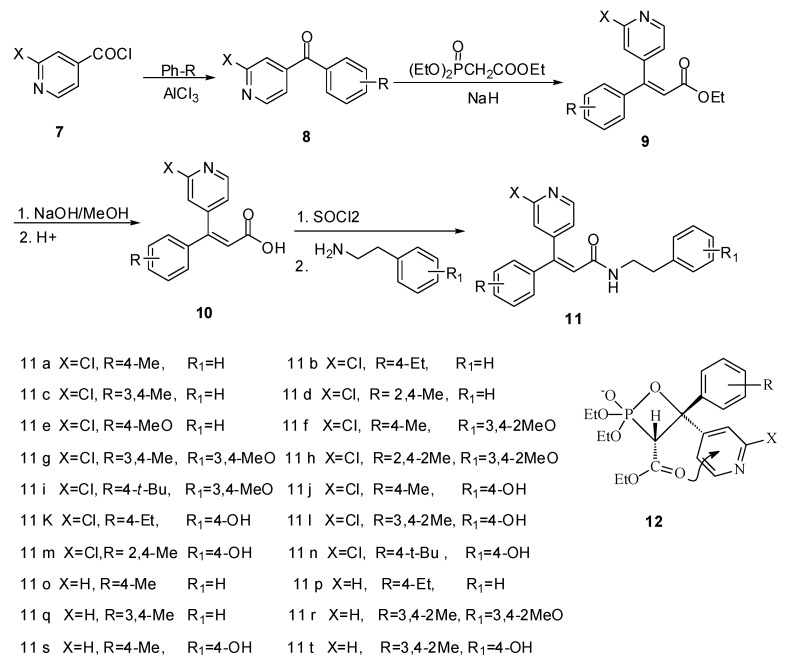
Synthetic route to the title compounds **11**.

**Figure 2:**
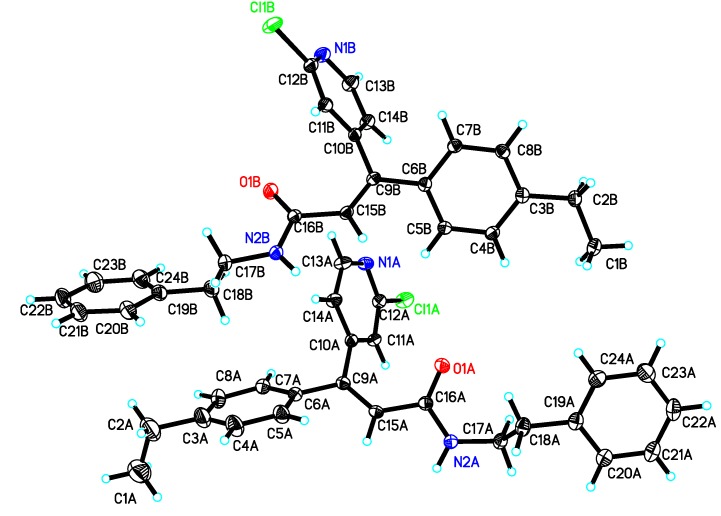
Crystal structure of **11b**.

The synthetic route to the title 20 compounds involves four-step reaction including Wittig-Horner reaction as the key step (Scheme 1). The Wittig-Horner reaction usually gives a mixture of *E*/*Z* isomers, but the ratio is quite different depending to the structure of substrates, reaction temperature, solvent, catalyst and so on. In our synthetic route, the reaction of compound **8** with ethyl diethoxyphosphinic acetate predominantly generated the more stable *cis*-intermediate **12** rather than the *trans-*form. Tis is attributed to the fact that in the *cis*-form the electron-rich carbonyl oxygen has a tendency to donate electrons to the electron-deficient pyridine ring, which leads to the formation of the more stable intermediate and the *cis-*product **9**. The deduction was confirmed by the crystal structure of **11b**, which was determined by X-ray diffraction analysis (Figure 2 and Table 1).

It can be seen from Figure 1 that the carbonyl and the pyridine ring bearing a chlorine atom are oriented towards the same side, so we can conclude that compound **11b** is the *Z*-isomer.

**Table 1:** Crystal structure and data refinement parameters.

Compound	11b
Empirical formula	C_24_H_23_ON_2_C
Formula weight	391.2
Crystal system/space groupOrthorhombic	Triclinic, P-1
a / Å	8.6568(17)
b / Å	13.927(3)
c / Å	18.507(4)
*α */ °	90°
*β */ °	90°
*γ */ °	90°
V / Å^3^	2056.1(7)
Z	4
D calc (g/cm^3^)	1.263
*μ *(mm^−1)^	0.202
Crystal size (mm)	0.50 × 0.36 × 0.36
Color/shape	Colorless/ rectangle
Temp (K)	173(2)K
Theta range for collection	2.39 < θ < 25.00°
Reflections collected	20946
Independent reflections	7232
Data/restraints/parameters	7232 / 0 / 505
Goodness of fit on F2	1.062
Final R indices [I *> *2*σ*(I)]	R_1_ = 0.0481, wR_2_ = 0.1187
R indices (all data)	R_1_ = 0.0514, wR_2_ = 0.1213
Largest difference peak/hole	0.686 and −0.312 e.A^−3^

### 2.2. Biological Activity

The fungicidal activity of the target compounds was tested *in vitro* against seven kinds of common plant pathogens including *R. solani*, *P. parasitica*, *B. cinerea*, *S. sclerotiorum*, *V. mali*, *P. asparagi and C. lindemuthianum). *As shown in Table 2, most of the title compounds at 50 μg·mL^−1^indicated moderate to high activity in the initial screening against the tested pathogens except *P. parasitica *and *V. mali*, and the inhibition ratios of **11a** and **11l** reached 90% against *R. solani*. The antifungal activity is basically in the following order: **11a** > **11o**, **11b** > **11p**, **11c**> **11q**, **11g**> **11r**, **11j** > **11s**, **11l** > **11t**. That is to say, compounds bearing a chlorine atom in the pyridine ring indicated higher inhibition rates against the seven fungi than those with no chlorine atom in the ring. The results also showed that the morpholine ring of the lead compound pyrimorph could be replaced by *β*-phenylethylamine.

As structural analogs of lansiumamide B (**6**), the nematicidal activity of title compounds was tested against *Bursaphelenchus xylophilus. *The data is presented in the form of mean mortality and corrected mortality (%) in Table 3. Next, the LD_50 _of compounds **11b**, **11c** and **11f** were determined and the results are listed in Table 4. 

The results of Table 3 and Table 4 indicated that the twenty novel cinnamide derivatives displayed moderate insectidal activity. Compound **11b** indicated the highest nematicidal activity, and the LC_50_ is 113.8 μg mL^−1^ against *B. xylophilus*. Compounds **11m** and **11q** also exhibited good mortality.

**Table 2:** Fungicidal activity of the title compounds (inhibition rate, %).

Compd.	*R. solani*	*P. parasitica*	*B. cinerea*	*S. sclerotiorum*	*V. mali*	*P. asparagi*	*C. lindemuthianum*
11a	93.88	24.41	44	37	48	59.70	59.7
11b	22.45	26.77	50	44	20	34.33	34.33
11c	76.77	1.61	67	72	52	35.82	55.97
11d	4.52	11.29	43	24	31	22.39	47.01
11e	13.55	−2.42	55	42	−6.3	31.34	43.28
11f	82.58	0	59	42	42	39.55	55.22
11g	23.81	14.17	33	28	36	23.88	23.88
11h	35.48	22.58	40	68	29	29.10	42.54
11i	14.84	−8.06	48	53	18	23.13	22.39
11j	72.9	−18.5	33	29	29	55.22	16.42
11k	67.74	28.23	37	17	24	32.09	24.63
11l	90.97	−18.5	54	44	−6.3	17.16	35.82
11m	13.55	−25	38	27	26	42.54	21.64
11n	9.03	−13.7	50	29	−7.8	32.84	39.55
11o	15.48	−17.7	43	16	36	44.03	32.84
11p	9.68	5.65	48	45	30	28.36	37.31
11q	3.87	−9.68	15	2.7	18	23.88	17.16
11r	5.81	−14.5	52	11	68	16.42	28.36
11s	10.32	−17.7	47	5.3	−2.3	28.36	28.36
11t	16.13	14.52	64	27	−2.3	55.97	38.81
pyrimorph	97.20	-	-	79	-	66.50	-
carbendazim	100	14.52	38	100	100		82.09
chlorothalonil	75.48	63.71	95	70	88	82.09	
azoxystrobin	89.03	37.9	80	100	92		

**Table 3:** Nematicidal Activity of the title compounds.

Compd.	24 h		48 h		72 h	
	Mortality (%)	Corrected mortality (%)	Mortality (%)	Corrected mortality (%)	Mortality (%)	Corrected mortality (%)
11a	21.67 ± 1.67	15.80 ± 1.79	5.00 ± 1.15	28.13 ± 1.28	45.00 ± 1.89	35.35 ± 0.39
11b	23.67 ± 0.88	17.95 ± 0.95	33.33 ± 1.67	26.29 ± 1.84	72.67 ± 1.20	67.87 ± 1.41
11c	20.67 ± 0.67	14.73 ± 0.72	29.00 ± 1.08	21.49 ± 1.30	53.33 ± 1.40	45.15 ± 1.82
11d	28.33 ± 1.20	22.97 ± 1.29	31.67 ± 1.67	24.44 ± 1.84	38.67 ± 1.86	27.91±1.18
11e	19.67 ± 0.33	13.65±0.36	44.67 ± 1.20	38.82 ± 1.33	50.67 ± 2.96	42.01 ± 1.48
11f	19.33 ± 0.67	13.29 ± 0.72	44.33 ± 0.88	38.45 ± 0.97	52.33 ± 1.33	43.97 ± 1.71
11g	15.00 ± 0.00	8.63 ± 0.00i	25.67 ± 1.33	17.81 ± 1.58	33.33 ± 0.88	21.64 ± 1.03
11h	24.67 ± 1.67	19.02 ± 1.87	40.33 ± 0.88	34.02 ± 0.98	47.00 ± 1.45	38.09 ± 1.71
11i	11.00 ± 0.57	4.33 ± 0.62	44.67 ± 0.67	38.82 ± 0.74	53.33 ± 1.67	45.15 ± 1.96
11j	14.00 ± 1.53	7.56 ± 1.64	21.00 ± 0.58	12.65 ± 0.64	27.00 ± 1.15	14.20 ± 1.36
11k	20.67 ± 0.67	14.73 ± 0.72	40.00 ± 1.15	33.60 ± 1.28	52.33 ± 1.45	43.97 ± 1.71
11l	10.67 ± 0.33	3.97 ± 0.36	23.00 ± 1.00	14.86 ± 1.11	35.33 ± 1.60	23.99 ± 1.06
11m	18.33 ± 1.02	12.22 ± 1.18	50.00 ± 1.15	44.71 ± 1.28	61.00 ± 1.08	54.16 ± 1.44
11n	26.67 ± 0.67	21.17 ± 0.95	34.67 ± 1.45	27.76 ± 1.61	41.67 ± 1.67	31.44 ± 1.96
11o	15.67 ± 0.67	9.34 ± 0.92	23.00 ± 1.00	14.86 ± 1.11	30.33 ± 0.33	18.11 ± 0.39
11p	14.00 ± 1.00	7.56 ± 1.07	17.00 ± 1.53	8.23 ± 1.69	22.33 ± 0.88	8.71 ± 1.04
11q	33.67 ± 0.88	28.70 ± 0.95	52.67 ± 1.20	47.67 ± 1.32	65.67 ± 1.67	49.07 ± 1.96
11r	1.67 ± 0.88	13.65 ± 0.95	24.33 ± 1.20	16.33 ± 1.33	31.33 ± 0.67	19.29 ± 0.79
11s	14.67 ± 1.45	8.27 ± 1.56	24.67 ± 1.45	16.70 ± 1.61	31.33 ± 1.33	19.29 ± 0.79
11t	18.00 ± 1.15	11.86 ± 1.24	30.00 ± 1.52	22.60 ± 1.78	42.33 ± 1.45	32.22 ± 1.71
6	100.00±0.00	100.00±0.00	100.00±0.00	100.00±0.00	100.00±0.00	100.00±0.00
CK	6.97 ± 1.06 ^2^		9.50 ± 1.05 ^2^		13.02 ± 0.88 ^2^	

Note. The corrected mortality = an average value ± standard error, which is the result after 72 hours of administration. In this column, the same character means no prominent difference at 5% level (LSD).

**Table 4:** Toxicity of selected compounds against *Bursaphelen-chus xylophilusat*.

Time	Compd.	Regression equation	Correlation coefficient	LD_50_ (mg/L)	95% Confidence limit (mg/L)
24 h	11b	Y = 0.41 + 1.85x	0.99	300.56	241.33–373.00
48 h		Y = 0.14 + 2.17x	0.99	173.77	165.11–182.88
72 h		Y = 0.59 + 2.14x	0.99	113.79	95.27–135.90
24 h	11c	y = 0.15 + 1.84x	0.97	429.66	285.32–646.99
48 h		y = 0.02 + 2.00x	0.98	314.58	231.32–429.57
72 h		y = 0.37 + 1.92x	0.95	252.36	170.43–373.67
24 h	11f	y = 0.74 + 1.71x	0.98	305.34	232.32–401.02
48 h		y = 1.89 + 1.38x	0.98	178.50	139.51–228.41
72 h		y = 2.28 + 1.30x	0.94	126.18	80.47–180.78
24 h	6	y = 0.26 + 5.70x	0.96	8.38	7.77–9.03
48 h		y = 0.44 + 5.67x	0.95	6.36	5.90–6.84
72 h		y = 0.62 + 6.00x	0.96	5.38	4.96–5.78
24 h	Avermectins	y = 2.58 + 1.75x	0.94	18.88	10.56–33.75
48 h		y = 3.35 + 1.73x	0.98	8.98	8.30–9.73
72 h		y = 3.27 + 2.00x	0.99	7.23	6.43–8.12

## 3. Experimental

### 3.1. General: Instruments and Methods

The melting points were determined on an XT-4 microscope melting point apparatus (Beijing Tech Instruments Co., Beijing, China) and are uncorrected. ^1^H-NMR spectra were obtained at 300 MHz using a Bruker Avance DPX 300 spectrometer in CDCl_3_ or DMSO-d_6_ solution, with tetramethylsilane as the internal standard. Chemical-shift values (δ) were given in parts per million (ppm). Mass spectra were obtained at Agilent 1100 series LC/MSD Trap. IR spectra data (cm^−1^) were determined on VECTOR2 (KBr). The determination of unit cell and data collection was performed on a Rigaku raxis Rapid IP Area Detector by using a graphite-monochromatized diffraction meter with Mo*Ka* radiation. Elemental analyses were carried out on an Elementar Vario EL instrument. All analytically pure reagents were purchased from Bailingwei or Beijing Chemical Reagent Co., and the solvents were dried by standard methods in advance and distilled before use.

### 3.2. General Synthetic Procedures for the Preparation of Compounds ***10a**–**10j***

A mixture of 70% sodium hydride (15 mmol) and tetrahydrofuran (THF, 10 mL) was added dropwise to the solution of ethyl diethoxyphosphinic acetate in THF (3 mL, 10 mmol) at 0 °C under stirring. After the evolution of hydrogen ceased, compound **9** in THF (6 mL, 5 mmol) was added dropwise and the mixture was stirred at r.t. for a further 10 h. Water (30 mL) was added slowly and the mixture was extracted with ethyl acetate (10 mL × 4). The combined organic phase was washed with water, dried with anhydrous magnesium sulphate, filtered, and evaporated to dryness *in vacuo* to give ethyl 3-(pyrid-4-yl)cinnamate as a colorless oil. The oil was dissolved in methanol (30 mL) and NaOH solution (7.5 mL, 2 mol/L), and was stirred at r.t. for further 10 h. The reaction mixture was evaporated to remove the solvent *in vacuo*, and the mixture was extracted twice with ethyl ether (20 mL) after water (40 mL) was added. The aqueous layer was then acidified to pH 2-3 and extracted with ethyl ether (20 mL × 3) again. The combined organic phase was treated successively by washing with water, drying with anhydrous Na_2_SO_4_, filtering, evaporating to dryness and recrystallizing from glacial HOAc, to give compounds **10** as white powders.

*3-(2-Chloropyridin-4-yl)-3-p-tolylacrylic acid *(**10a**, C_15_H_12_O_2_NCl, X = Cl, R = 4-Me). Yield 91%, Mp. 191~192 °C. ^1^H-NMR (CDCl_3_), δ 2.30 (s, 3H, CH_3_), 6.50 (s, 1H, =CH), 7.17–7.34 (m, 6H, Ar-H), 8.38(d, *J* = 5.07 Hz, 1H, pyridine-H), 12.36 (brs, 1H, OH).

*3-(2-Chloropyridin-4-yl)-3-(4-ethylphenyl) acrylic acid *(**10b**, C_16_H_14_O_2_NCl,X = Cl, R = 4-Et). Yield 72%, Mp. 152~153 °C. ^1^H-NMR (CDCl_3_), δ 1.21 (m, 3H, 2CH_3_), 2.59 (m, 2H, CH_2_), 6.50 (s, 1H, =CH), 7.08–7.35 (m, 6H), 8.38–8.44 (m, 1H, pyridine-H), 12.40 (brs, 1H, OH).

*3-(2-Chloropyridin-4-yl)-3-(3,4-dimethylphenyl) acrylic acid* (**10c**, C_16_H_14_O_2_NCl, X = Cl, R = 3,4-Me). Yield 96%, Mp. 201~202 °C. ^1^H-NMR (CDCl_3_), δ2.20 (s, 6H, ), 6.61 (s, 1H, =CH), 6.98–7.17 (m, 3H, Ph-H), 7.81–7.83 (dd, 2H, pyridine-H), 8.89 (d, *J* = 1.29 Hz, 1H, pyridine-H), 8.92 (s, 1H, OH).

*3-(2-Chloropyridin-4-yl)-3-(2,4-dimethylphenyl) acrylic acid *(**10d**, C_16_H_14_O_2_NCl, X = Cl, R = 2,4-Me). Yield 85%, Mp. 189~191 °C. ^1^H-NMR (CDCl_3_), δ2.10 (s, 3H, CH_3_), 2.13 (s, 3H, CH_3_), 6.63 (s, 1H, =CH), 6.98 (d, *J* = 2.03 Hz, 1H), 7.01–7.18 (m, 4H, Ph-H), 7.84 (dd, 2H, pyridine-H), 8.75 (d, *J* = 3.57 Hz, 1H), 12.38 (brs, 1H, OH).

*3-(2-Chloropyridin-4-yl)-3-(4-methoxyphenyl) acrylic acid *(**10e**, C_16_H_14_O_3_NCl, X = Cl, R = 4-OMe). Yield 68%, Mp. 168~170 °C. ^1^H-NMR(CDCl_3_), δ 3.80 (s, 3H, CH_3_), 6.51 (s, 1H, =CH), 7.06–7.22 (m, 4H, Ph-H), 7.40 (dd, 2H, Pyridine-H), 8.75 (d, *J* = 3.57 Hz, 1H, Pyridine-H), 12.46 (brs, 1H, OH).

*3-(4-tert-Butylphenyl)-3-(2-chloropyridin-4-yl)-acrylic acid* (**10f**, C_17_H_16_O_2_NCl, X = Cl, R = 4-*t*-Bu). Yield 55%, Mp. 176~177 °C. ^1^H-NMR(CDCl_3_), δ 1.32 (s, 9H, 3CH_3_), 6.23 (s, 1H, =CH), 7.08–7.20 (m, 4H, Ph-H), 7.40 (dd, 2H, Pyridine-H), 8.45 (d, *J* = 5.01 Hz, 1H, Pyridine-H), 12.36 (brs, 1H, OH).

*3-(Pyridin-4-yl)-3-p-tolylacrylic acid *(**10g**, C_15_H_13_O_2_N, X = H, R = 4-Me). Yield 83%, Mp. 221~222 °C. ^1^H-NMR (CDCl_3_), δ 2.40 (s, 3H, CH_3_), 6.48 (s, 1H, =CH), 7.09–7.23 (m, 6H, Ar-H), 8.55–8.60 (m, 2H, pyridine-H), 11.89 (brs, 1H, OH).

*3-(4-Ethylphenyl)-3-(pyridin-4-yl)-acrylic acid *(**10h**, C_16_H_15_O_2_N, X = H, R = 4-Et). Yield 59%, Mp. 205~206 °C. ^1^H-NMR (CDCl_3_), δ 1.14–1.25 (m, 3H, CH_3_), 2.50–2.67 (m, 2H, CH_2_), 6.48 (s, 1H, =CH), 6.90–7.14 (m, 4H), 7.20–7.25 (m, 2H), 11.89 (brs, 1H, OH).

*3-(3,4-Dimethylphenyl)-3-(pyridin-4-yl)acrylic acid *(**10i**, C_16_H_15_O_2_N, X = H, R = 3,4-Me). Yield 77%, Mp. 238~240 °C. ^1^H-NMR (CDCl_3_), δ 2.20 (d, *J* = 14.55 Hz, 3H, CH_3_), 2.27 (d, *J* = 3.54 Hz, 3H, CH_3_), 6.48 (s, 1H, =CH), 6.90–7.14 (m, 3H, Ph-H), 7.20–7.25 (m, 2H, pyridine-H), 8.52–8.58 (m, 2H, pyridine-H), 11.90 (brs, 1H, OH).

*3-(2,4-Dimethylphenyl)-3-(pyridin-4-yl) acrylic acid* (**10j**, C_16_H_15_O_2_N, X = H, R = 2,4-2Me). Yield 62%, Mp. 207~208 °C. ^1^H-NMR (CDCl_3_), δ 2.05 (s, 3H, CH_3_), 2.33 (s, 3H, CH_3_), 6.20 (s, 1H, =CH), 7.00–7.08 (m, 3H, Ph-H), 7.30 (dd, 2H, pyridine-H), 8.52 (dd, 2H, pyridine-H), 9.99 (brs, 1H, OH).

### 3.3. General Synthetic Procedures for Compounds ***11a**–**11t***

Thionyl chloride (6.95 mmol) was added dropwise to the mixture of compound **10 **(3.66 mmol) and DMF of catalytic amount in CH_2_Cl_2_ (20 mL) and then stirred continuously for eight hours at 0 °C. The reaction mixture was evaporated to remove the solvent and excess thionyl chloride *in vacuo* and the residue was dissolve using CH_2_Cl_2_ (20 mL). *β*-Phenylethamine (4.13 mmol) and triethylamine (catalytic amount) in CH_2_Cl_2_ (10 mL) was added to the residue at 0 °C. The reaction mixture was stirred at r.t. for further 10 h, and then was washed with hydrogen chloride (1 mol/L), saturated potassium carbonate solution and water, dried over anhydrous MgSO_4_, and concentrated to give the crude product. Recrystallization from methanol afforded the title compounds. 

*3-(2-Chloropyridin-4-yl)-N-phenethyl-3-p-tolylacrylamide* (**11a**, X = Cl, R = 4-Me, R_1_ = H), Yield 59%, Mp. 158–160 °C,^1^H-NMR (CDCl_3_), δ 2.35 (s, 3H, CH_3_), 2.74 (t, 2H, CH_2_), 3.51(q, 2H, CH_2_), 5.60 (brs, 1H, NH), 6.28 (s, 1H, =CH), 7.04–7.33 (m, 11H, Ar-H), 8.37 (dd, 1H, *J *= 6.87, 6.40 Hz, pyridine-H). MS *m/z* (ESI) 377.2 [M+H]^+^ Anal. Calc. for C_23_H_21_ON_2_Cl (376.13): C, 73.30; H, 5.62; N, 7.43; found:C, 73.22; H, 5.62; N, 7.50.

*3-(2-Chloropyridin-4-yl)-3-(4-ethylphenyl)-N-phenethylacrylamide* (**11b**, X = Cl, R = 4-Et, R_1_ = H). Yield 88%, Mp. 142–143 °C, ^1^H-NMR (CDCl_3_), δ 1.23 (t, 3H, CH_3_), 2.65 (q, 2H, CH_2_), 2.74 (t, 2H, CH_2_), 3.50 (q, 2H, CH_2_), 5.60 (brs, 1H, NH), 6.29 (s, 1H, =CH), 7.07–7.33 (m, 11H, Ar-H), 8.37 (dd, *J *= 6.78. 6.39 Hz, 1H, pyridine-H ). MS *m/z* (ESI) 391.2[M+H]^+^. Anal. Calc. for C_24_H_23_ON_2_Cl (390.15): C, 73.74; H, 5.93; N, 7.17; found C, 73.61; H 5.84; N 7.24.

*3-(2-Chloropyridin-4-yl)-3-(3,4-dimethylphenyl)-N-phenethylacrylamide* (**11c**, X = Cl, R = 3,4-Me, R_1_ = H). Yield 93%, Mp. 162–163 °C, ^1^H-NMR(CDCl_3_), δ 2.21 (q, 3H, CH_3_), 2.26 (t, 3H, CH_3_), 2.74 (t, 2H, CH_2_), 3.50 (q, 2 H, CH_2_), 5.60 (brs, 1H, NH), 6.28 (s, 1H, =CH), 6.87–7.33 (m, 10H, Ar-H), 8.33 (dd, *J *= 6.63, 6.81 Hz, 1H, pyridine-H).MS *m/z* (ESI) 391.2 [M+H]^+^. Anal. Calc. For C_24_H_23_ON_2_Cl (390.15): C, 73.73; H, 5.93; N 7.17; found: C 73.74; H, 5.93; N, 7.20.

*3-(2-Chloropyridine-4-yl)-3-(2,4-dimethylphenyl)acrylamide* (**11d**, X = Cl, R = 2,4-2Me, R_1_ = H). Yield 68%, Mp. 160–161 °C, ^1^H-NMR (CDCl_3_), δ 2.01 (q, 3H, CH_3_), 2.30 (t, 3H, CH_3_), 2.77 (t, 2H, CH_2_), 3.53 (q, 2H, CH_2_), 5.45 (brs, 1H, NH), 6.05 (s, 1H, =CH), 6.98–7.32 (m, 10H, Ar-H), 8.33 (dd, *J *= 7.5, 7.5 Hz, 1H, pyridine-H). MS *m/z* (ESI) 391.20 [M+H]^+^. Anal. Calc. for C_24_H_23_ON_2_Cl (390.15): C, 73.74; H 5.93; N, 7.17;. found: C, 73.72; H, 5.93; N, 7.22.

*3-(2-Chloropyridin-4-yl)-3-(4-methoxyphenyl)-N-phenethylacrylamide*** (11e**, X = Cl, R = 4-MeO, R_1_ = H). Yield 71%, Mp. 185–186 °C, ^1^H-NMR (CDCl_3_), δ 2.74 (t, 2H, CH_2_), 3.50 (q, 2H, CH_2_), 3.80 (s, 3H, CH_3_O), 5.45 (brs, 1H, NH), 6.24 (s, 1H, =CH), 6.83–7.33 (m, 11H, Ar-H), 8.37 (dd, *J *= 5.70. 5.67 Hz, 1H, pyridine-H). MS*m/z* (ESI) 393.13[M+H]^+^. Anal. Calc. for C_23_H_21_O_2_N_2_Cl (392.13): C, 70.71; H, 5.39; N, 7.13; found：C, 70.32; H, 5.34; N, 7.20.

*3-(2-Chloropyridin-4-yl)-N-(3,4-dimethoxyphenethyl)-3-p-tolylacrylamide* (**11f**, X = Cl, R = 4-Me, R_1_ = 3,4-MeO). Yield 77%, Mp. 181–182 °C, ^1^H-NMR (CDCl_3_), δ 2.30 (t, 3H, CH_3_), 2.36 (q, 2H, CH_2_), 3.21 (q, 2H, CH_2_), 3.71 (s, 3H, CH_3_O), 3.73 (s, 3H, CH_3_O), 6.58 (s, 1H, =CH), 6.67–7.25 (m, 9H, Ar-H), 8.23 (brs, 1H, NH), 8.38 (dd, *J *= 6.54, 6.54 Hz, 1H, pyridine-H). MS *m/z* (ESI) 437.2 [M+H]^+^. Anal. Calc. for C_25_H_25_O_3_N_2_Cl (436.16): C, 68.72; H, 5.77; N, 6.41; found: C, 68.30, H, 5.78; N, 6.35. 

*3-(2-Chloropyridin-4-yl)-N-(3,4-dimethoxyphenethyl)-3-p-tolylacrylamide* (**11f**, X = Cl, R = 4-Me, R_1_ = 3,4-MeO). Yield 77%, Mp. 181–182 °C, ^1^H-NMR (CDCl_3_), δ 2.30 (t, 3H, CH_3_), 2.36 (q, 2H, CH_2_), 3.21 (q, 2H, CH_2_), 3.71 (s, 3H, CH_3_O), 3.73 (s, 3H, CH_3_O), 6.58 (s, 1H, =CH), 6.67-7.25 (m, 9H, Ar-H), 8.23 (brs, 1H, NH), 8.38 (dd, *J *= 6.54, 6.54 Hz, 1H, pyridine-H). MS *m/z* (ESI) 437.2 [M+H]^+^. Anal. Calc. for C_25_H_25_O_3_N_2_Cl (436.16): C, 68.72; H, 5.77; N, 6.41; found: C, 68.30, H, 5.78; N, 6.35. 

*3-(2-Chloropyridin-4-yl)-N-(3,4-dimethoxyphenethyl)-3-(3,4-dimethylphenyl)acrylamide* (**11g**, X = Cl, R = 3,4-Me, R_1_ = 3,4-MeO). Yield 81%, Mp. 157–159 °C, ^1^H-NMR (CDCl_3_), δ 2.20 (s, 3H, CH_3_), 2.26 (s, 3H, CH_3_), 2.68 (t, 2H, CH_2_), 3.52 (q, 2H, CH_2_), 3.85 (s, 3H, CH_3_O), 3.87 (s, 3H, CH_3_O), 5.50 (brs, 1H, NH), 6.28 (s, 1H, =CH), 6.62–7.18 (m, 8H, Ar-H), 8.38 (dd, *J *= 5.4, 4.8 Hz, 1H, pyridine-H). MS *m/z* (ESI) 451.2 [M+H]^+^. Anal. Calc. for C_26_H_27_O_3_N_2_Cl (450.17): C, 69.25; H, 6.03; N, 6.21; found,: C, 69.01; H, 5.99; N, 6.20.

*3-(2-Chloropyridin-4-yl)-N-(3,4-dimethoxyphenethyl)-3-(2,4-dimethylphenyl)acrylamide* (**11h**, X = Cl, R = 2,4-Me, R_1_ = 3,4-MeO). Yield 77%, Mp. 111–113 °C, ^1^H-NMR (CDCl_3_), δ 1.99 (d, 3H, CH_3_), 2.02 (d, 3H, CH_3_), 2.34 (t, 2H, CH_2_), 3.51 (q, 2H, CH_2_), 3.84 (s, 3H, CH_3_O), 3.85 (s, H, CH_3_O), 5.50 (brs, 1H, NH), 6.05 (s, 1H, =CH), 6.58–7.14 (m, 8H, Ar-H), 8.39 (dd, *J *= 6.18, 6.39 Hz, 1H, pyridine-H). MS *m/z* (ESI) 451.2 [M+H]^+^. Anal. Calc. for C_26_H_27_O_3_N_2_Cl (450.17): C, 69.25; H, 6.03; N, 6.21; found: C, 69.10; H, 6.03; N, 6.20.

*3-(4-tert-Butylphenyl)-3-(2-chloropyridin-4-yl)-N-(3,4-dimethoxyphenethyl)acrylamide* (**11i**, X=Cl, R=4-*t*-Bu, R_1_ = 3,4-2MeO). Yield 53%, Mp. 146–148 °C, ^1^H-NMR (CDCl_3_), δ 1.33 (t, 9H, C(CH_3_)_3_), 2.70 (t, 2H, CH_2_), 3.48 (q, 2H, CH_2_), 3.84 (s, 3H, CH_3_O), 3.86 (s, 3H, CH_3_O), 5.50 (brs, 1H, NH), 5.57 (t, 1 H, =CH), 6.63–7.38 (m, 9H, Ar-H ), 8.38 (dd, *J *= 7.02, 6.93 Hz, 1H, pyridine-H). MS *m/z* (ESI) 479.20[M+H]^+^. Anal. Calc. for C_28_H_31_O_3_N_2_Cl (478.20): C, 70.21; H, 6.52; N, 5.85;. found: C, 69.90; H, 6.50; N, 5.90.

*3-(2-Chloropyridin-4-yl)-N-(4-hydroxyphenethyl)-3-p-tolylacrylamide* (**11j**, X = Cl, R = 4-Me, R_1_ = 4-OH). Yield 94%, Mp. 233–234 °C, ^1^H-NMR (DMSO), δ 2.30 (s, 3H, CH_3_), 2.36 (s, 3H, CH_3_), 2.51 (t, 2H, CH_2_), 3.21 (t, 2H, CH_2_), 6.56 (s, 1H, =CH), 6.63–7.29 (m, 8H, Ar-H), 7.17–7.30 (m, 2H, pyridine-H), 8.07 (brs, 1H, NH), 8.38 (d, *J *= 5.25 Hz, 1H, pyridine-H), 9.18 (brs, 1H, OH). MS *m/z* (ESI) 391.2 [M-H]^+^ Anal. Calc. for C_23_H_21_O_2_N_2_Cl (392.13): C, 70.31; H, 5.39; N, 7.13; found: C, 69.95; H, 5.33; N, 7.16.

*3-(2-Chloropyridin-4-yl)-3-(4-ethylphenyl)-N-(4-hydroxyphenethyl)acrylamide* (**11k**, X = Cl, R = 4-Et, R_1_ = 4-OH). Yield 88%, Mp. 231–232 °C, ^1^H-NMR (DMSO), δ 1.15–1.23 (m, 3H, CH_3_), 2.45–2.52 (m, 2H, CH_2_), 2.60–2.67 (m, 2H, CH_2_), 3.15–3.22 (m, 2H, CH_2_), 6.64 (s, 1H, =CH), 6.65–7.29 (m, 8H, Ar-H), 7.17–7.30 (m, 2H, pyridine-H), 8.06 (brs, 1H, NH), 8.38 (d, *J *= 5.25 Hz, 1H, pyridine-H) 9.18 (s, 1 H, OH). MS *m/z* (ESI) 405.20 [M-H]^+^ Anal. Calc. for C_24_H_23_O_2_N_2_Cl (406.14): C, 70.84; H, 5.70; N, 6.88; found: C, 70.57; H, 5.70; N, 6.81.

*3-(2-Chloropyridin-4-yl)-3-(3,4-dimethylphenyl)-N-(4-hydroxyphenethyl)acrylamide *(**11l**, X = Cl, R = 3,4-Me, R_1_ = 4-OH). Yield 95%, Mp. 159–161 °C, ^1^H-NMR (DMSO), δ 2.27 (s, 3H, -CH_3_), 2.30 (s, 3H, -CH_3_), 2.51 (t, 2H, -CH_2_), 3.19 (t, 2H, -CH_2_), 5.76 (s, 1H, =CH), 6.54–7.21 (m, 9H, Ar-H), 8.21(brs, 1H, -NH), 8.36 (d, 1H, *J *= 5.07 Hz, pyridine-H), 9.18 (brs, 1H, OH). MS *m/z* (ESI) 405.3 [M-H]^+^ Anal. Calc. for C_24_H_23_O_2_N_2_Cl (406.14): C, 70.84; H, 5.70; N, 6.88; found: C, 70.59; H, 5.70, N, 6.78.

*3-(2-Chloropyridin-4-yl)-3-(2,4-dimethylphenyl)-N-(4-hydroxyphenethyl)acrylamide *(**11m**, X = Cl, R = 2,4-Me, R_1_ = 4-OH). Yield 89%, Mp. 244–246 °C, ^1^H-NMR (DMSO), δ 2.04 (s, 6H, -CH_3_), 2.56 (t, 2H, -CH_2_), 3.21 (t, 2H, -CH_2_), 6.14 (s, 1H, =CH), 6.66–7.19 (m, 9H, Ar-H ), 8.26 (brs, 1H, -NH), 8.31 (dd, *J* = 5.4, 5.1Hz, 1 H, pyridine-H), 9.10 (brs, 1H, -OH). MS *m/z* (ESI) 405.1 [M-H]^+^. Anal. Calc. for C_24_H_2_3O_2_N_2_Cl (406.14): C, 70.84; H, 5.70; N, 6.88; found: C, 70.53; H, 5.60; N, 6.79.

*3-(4-tert-Butylphenyl)-3-(2-chloropyridin-4-yl)-N-(4-hydroxyphenethyl)acrylamide* (**11n**, X = Cl, R = 4-*t*-Bu, R_1_ = 4-OH). Yield 45%, Mp. 205–207 °C, ^1^H-NMR (DMSO), δ 1.28 (s, 9H, 3CH_3_), 2.50 (t, 2H, CH_2_), 3.21 (t, 2H, CH_2_), 6.57 (s, 1H, =CH), 6.63–7.95 (m, 10H, Ar-H), 8.23 (brs, 1H, NH), 8.37 (dd, *J *= 6.90, 6.60 Hz, 1H, pyridine-H), 9.19 (brs, 1H, OH).MS *m/z* (ESI) 433.20 [M-H]^+^. Anal. Calc. for C_26_H_2_7O_2_N_2_Cl (434.18): C, 71.80; H, 6.26; N, 6.44; found: C, 71.54; H, 6.03; N, 6.21.

*N-phenethyl-3-(pyridin-4-yl)-3-p-tolylacrylamide* (**11o**, X = H, R = 4-Me, R_1_ = H). Yield 58%, Mp. 191–192 °C, ^1^H-NMR (DMSO), δ 2.35 (s, 3H, CH_3_), 2.70 (t, 2H, -CH_2_), 3.46 (m, 2H, -CH_2_), 5.42 (brs, 1H, -NH), 6.32 (s, 1H, =CH), 7.06–7.31 (m, 11H, Ar-H), 8.60 (m, 2H, pyridine-H). MS *m/z* (ESI) 343.20[M+H]^+^. Anal. Calc. for C_23_H_22_ON_2 _(342.17): C, 80.67; H, 6.48; N, 8.18; found: C, 80.63; H, 6.46; N, 8.26.

*3-(4-Ethylphenyl)-N-phenethyl-3-(pyridin-4-yl)acrylamide* (**11p**, X = H, R = 4-Et, R_1_ = H). Yield 56%, Mp. 135–137 °C, ^1^H-NMR (CDCl_3_), δ 1.21 (t, 3H, CH_3_), 2.36 (q, 2H, CH_2_), 2.63 (t, 2H, CH_2_), 3.46 (q, 2H, CH_2_), 5.41 (brs, 1H, NH), 6.32 (s, 1H, =CH), 7.06–7.31 (m, 12H, Ar-H), 8.61 (dd, *J *= 1.65, 1.65 Hz, 2H, pyridine-H). MS *m/z* (ESI) 357.20 [M+H]^+^. Anal. Calc. for C_24_H_24_ON_2_ (356.19): C, 80.87; H, 6.79; N, 7.86; found: C, 80.73; H, 6.80; N, 7.89.

*3-(3,4-Dimethylphenyl)-N-phenethyl-3-(pyridin-4-yl)acrylamide* (**11q**, X = H, R = 3,4-Me, R_1 _= H). Yield 93%, Mp. 182–183 °C, ^1^H-NMR (CDCl_3_), δ 2.21 (t, 3H, CH_3_), 2.36 (q, 3H, CH_3_), 2.63 (t, 2H, CH_2_), 3.46 (m, 2H, CH_2_), 5.41 (brs, 1H, NH), 6.32 (s, 1H, =CH), 7.06–7.31 (m, 11H, Ar-H), 8.54 (dd, *J *= 1.59, 1.59 Hz, 1H, pyridine-H). MS *m/z* (ESI) 357.20 [M+H]^+^. Anal. Calc. for C_24_H_24_ON_2_ (356.19): C, 80.87; H, 6.79; N, 7.86; found: C, 80.85; H, 6.78; N, 7.90.

*N-(3,4-Dimethoxyphenethyl)-3-(3,4-dimethylphenyl)-3-(pyridin-4-yl)acrylamide* (**11r**, X = H, R = 3,4-Me, R_1_ = 3,4-2MeO). Yield 85%, Mp. 172–174 °C, ^1^H-NMR (CDCl_3_), δ 2.21 (t, 3H, CH_3_), 2.26 (s, 3H, CH_3_), 2.64 (t, 2H, CH_2_), 3.43 (q, 2H, CH_2_), 3.86 (s, 3H, CH_3_O), 3.87 (s, 3H, CH_3_O), 5.44 (brs, 1H, NH), 6.31 (s, 1H, =CH), 6.59–7.27 (m, 8H, Ar-H), 8.61 (dd, 2H, *J* = 1.53, 1.53 Hz, 2 H, pyridine-H). MS *m/z* (ESI) 417.30[M+H]^+^. Anal. Calc. for C_26_H_28_O_3_N_2_ (416.21): C, 74.97；H, 6.78; N, 6.73; found: C, 74.60; H, 6.80; N, 6.63.

*N-(4-Hydroxyphenethyl)-3-(pyridin-4-yl)-3-p-tolylacrylamide* (**11s**, X = H, R = 4-Me, R_1_ = 4-OH). Yield 88%, Mp. 244–246 °C, ^1^H-NMR (DMSO), δ 2.30 (s, 3H, CH_3_), 2.50–2.53 (m, 2H, CH_2_), 3.15–3.22 (m, 2H, CH_2_), 6.51 (s, 1H, =CH), 6.66–7.20 (m, 11H, Ar-H), 8.14 (brs, 1H, NH), 8.51–8.55 (m, 1H, pyridine-H), 9.19 (brs, 1H, OH). MS *m/z* (ESI) 356.20[M-2H]^+^. Anal. Calc. for C_23_H_22_O_2_N_2_ (358.20): C, 77.07; H, 6.19; N, 7.93; found: C, 76.93; H, 6.17; N, 7.71.

*3-(3,4-Dimethylphenyl)-N-(4-hydroxyphenethyl)-3-(pyridin-4-yl)acrylamide* (**11t**, X = H, R = 3,4-Me, R_1_ = 4-OH). Yield 96%, Mp. 159–161 °C, ^1^H-NMR (DMSO), δ 2.01 (s, 3H, CH_3_), 2.11 (s, 3H, CH_3_)_, _2.50–2.53 (t, 2H, CH_2_), 3.15–3.22 (q, 2H, CH_2_), 6.70 (s, 1H, =CH), 6.66–7.20 (m, 10H, Ar-H), 8.23 (brs, 1H, NH), 8.51–8.55 (m, 1H, pyridine-H), 9.20 (brs, 1H, OH). MS *m/z* (ESI) 370.20 [M-2H]^+^. Anal. Calc. for C_24_H_24_O_2_N_2 _(372.18): C, 77.39; H, 6.49; N, 7.52; found: C, 77.29; H, 6.44; N, 7.55.

Crystal data can be obtained from the Crystallographic Data Centre as supplementary publication number CCDC 848237. Copies of the data can be obtained, free of charge, on application to CCDC, 12 Union Road, Cambridge CB2 1EZ, UK. Fax: +44 1223 336033 or e-mail: deposit@ccdc.cam.ac.uk.

### 3.4. Bioassays

#### 3.4.1. Fungicidal Activity Test

The fungicidal activity of the target compounds against *R. solani*, *P. parasitica*, *B. cinerea*, *S. sclerotiorum*, *V. mali*, *P. asparagi and C. lindemuthianum * were evaluated using the mycelium growth rate test [21]. Pyrimorph, carbendazim, chlorothalonil, azoxystrobin were used as controls.Their relative inhibition ratio (%) h were calculated as equal to the (colony diameter of control − colony diameter of treatment)/(colony diameter of control − mycelial disks diameter) × 100. This experiment was conducted twice with four replicates.

#### 3.4.2. Nematicidal Activity

All the compounds were dissolved in acetone and diluted to a 200 mg L^−1^ solution with distilled water. The solution (1.5 mL) was placed in a 24 holes cellplate with nematode solution (0.5 mL, nearly five hundred nematodes of mixed ages), and the plate was put in a culture box at 25 °C. A distilled water test was used as blank control and every test was replicated three times. The mortality of insects was counted after 24, 48 and 72 hours of administration, and Abbotts formula was used to correct the mortality relative to that of negative control.

## 4. Conclusions

Using pyrimorph as the lead compound, we designed and synthesized twenty novel cinnamamides derivatives using Wittig-Horner reaction as the key step. X-ray data showed that the Wittig-Horner reactions mainly gave the *Z*-isomer product. The preliminary bioassay results demonstrated that most of the title compounds show a wide spectrum of activity against plant pathogens at 50 μg·mL^−1^. Compounds **11a** and **11l** showed higher fungicidal activity than the other compounds. The title compounds exhibited moderate nematicidal activity. Generally, the morpholine ring might be replaced by other amines and a chlorine atom in the pyridine ring is helpful to fungicidal activity.
